# SUMO-1 Modification on K166 of PolyQ-Expanded aTaxin-3 Strengthens Its Stability and Increases Its Cytotoxicity

**DOI:** 10.1371/journal.pone.0054214

**Published:** 2013-01-31

**Authors:** Ya-Fang Zhou, Shu-Sheng Liao, Ying-Ying Luo, Jian-Guang Tang, Jun-Ling Wang, Li-Fang Lei, Jing-Wei Chi, Juan Du, Hong Jiang, Kun Xia, Bei-Sha Tang, Lu Shen

**Affiliations:** 1 Department of Neurology, Xiangya Hospital, Central South University, Changsha, China; 2 Department of Neurology, the Second Xiangya Hospital, Central South University, Changsha, China; 3 National Laboratory of Medical Genetics of China, Central South University, Changsha, China; 4 Neurodegenerative Disorders Research Center, Central South University, Changsha, China; Stanford University School of Medicine, United States of America

## Abstract

Post-translational modification by SUMO was proposed to modulate the pathogenesis of several neurodegenerative diseases. Spinocerebellar ataxia type 3/Machado-Joseph disease (SCA3/MJD) is an autosomal dominant neurodegenerative disease caused by polyQ-expanded ataxin-3. We have previously shown that ataxin-3 was a new target of SUMOylation *in vitro* and *in vivo*. Here we identified that the major SUMO-1 binding site was located on lysine 166. SUMOylation did not influence the subcellular localization, ubiquitination or aggregates formation of mutant-type ataxin-3, but partially increased its stability and the cell apoptosis. Our findings revealed the role of ataxin-3 SUMOylation in SCA3/MJD pathogenesis.

## Introduction

Spinocerebellar ataxia type 3, also known as Machado-Joseph disease (SCA3/MJD), is the most common dominantly inherited ataxia [1]. It is a member of the polyglutamine (polyQ) neurodegenerative disease family which includes Huntington's disease (HD), spinal and bulbar muscular atrophy (SBMA), dentatorubral- pallidoluysian atrophy (DRPLA), and spinocerebellar ataxias 1, 2, 3, 6, 7, and 17 [2]–[4]. It has been demonstrated that polyQ expansion increased the cellular toxicity of the proteins and was responsible for the diseases. In normal individuals, the length of the CAG repeat varies between 12 and 37 trinucleotides whereas in SCA3/MJD patients it varies between 49 to 86 repeat units which located near the carboxy-terminus of SCA3 gene (MJD1) on chromosome 14q32.1 [5], leading to the toxic translational product of polyQ-expanded ataxin-3. The pathology of SCA3/MJD includes severe neuronal loss in the spinal cord and specific brain regions, such as dentate nuclei (cerebellum), pontine nuclei (brainstem), and substantia nigra (basal ganglia) [6]–[7]. Nuclear inclusions are detected in both affected and unaffected neurons of SCA3/MJD patients [8]–[9]. It is unclear if these aggregates contribute to neuronal dysfunction or possibly represent a protective mechanism, although some recent models suggest an inverse correlation between accumulation of aggregates and neuronal loss [10]–[11].

Recently, post-translational modifications have been shown to play a major role in the pathogenesis of polyQ diseases. There is increasing evidence demonstrating that different target proteins can be post-translational modified by SUMOylation. And the modified proteins are possible to involve in numerous neurological diseases including polyQ disorders [12]. SUMO is an ubiquitin-like protein with 20% identity to ubiquitin [13]. In vertebrates, the SUMO family has at least four members, SUMO-1, SUMO-2, SUMO-3, and SUMO-4 [14]–[17]. SUMO modification may have altered the function, activity or localization of its substrates [14], [18]–[20]. The conjugation of SUMO proteins, or SUMOylation, is a post-translational modification process that shares common ancestry and core enzymological features with ubiquitination but has distinct functional roles. SUMOs initially exist in an inactive form, which is processed by the SUMO specific protease to expose the glycine residues at their carboxy-terminal that are required for the formation of SUMO–protein conjugates. SUMOylation is a multistep process, which involves an activating enzyme E1 (SAE1 and SAE2), a conjugating enzyme E2 (Ubc9) and, in some cases, a ligating enzyme E3 [21]–[22].

SUMOylation is thought to modify the interactions in multiprotein complexes [23]. Beside its role as a covalent modifier, SUMO can bind non-covalently to SUMO-interacting motifs, which have been identified in many proteins [24], among which several are related to polyQ diseases such as androgen receptor, huntingtin, ataxin-1, and ataxin-7 [25]–[28]. SUMO and ubiquitin share a common three-dimensional structure, except that SUMO has an additional short amino terminal extension [29]. It has been reported that SUMO modification of some proteins on a lysine residue blocks ubiquitination at the same site, resulting in an inhibition of protein degradation and an alteration of protein function [26], [30]. In HD, SUMOylation of mutant huntingtin increases the stability of the protein and exacerbate neurodegeneration.

In our previous study, SUMO-1 had been identified as a novel ataxin-3-interacting protein by yeast two-hybrid technology. Both co-immunoprecipitation and immunofluorescence staining results proved that ataxin-3 was a target for SUMOylation both *in vitro* and *in vivo* [31], [32]. In order to reveal the exact role of SUMOylation in the pathogenesis of SCA3/MJD, here we report that the major SUMO-1 binding site was identified, which located on lysine 166 (K166) of the mutant-type ataxin-3. SUMOylation did not influence the subcellular localization, ubiquitination or aggregates formation of mutant-type ataxin-3, but partially increased its stability and the apoptosis rate of the cells. Our findings are the first to indicate the effect of SUMOylation on the stability and cellular toxicity of mutant ataxin-3 and implicate the role of SUMOylation in SCA3/MJD pathogenesis.

## Results

### Ataxin-3 was modified by SUMO-1 on lysine 166

**Figure 1:**
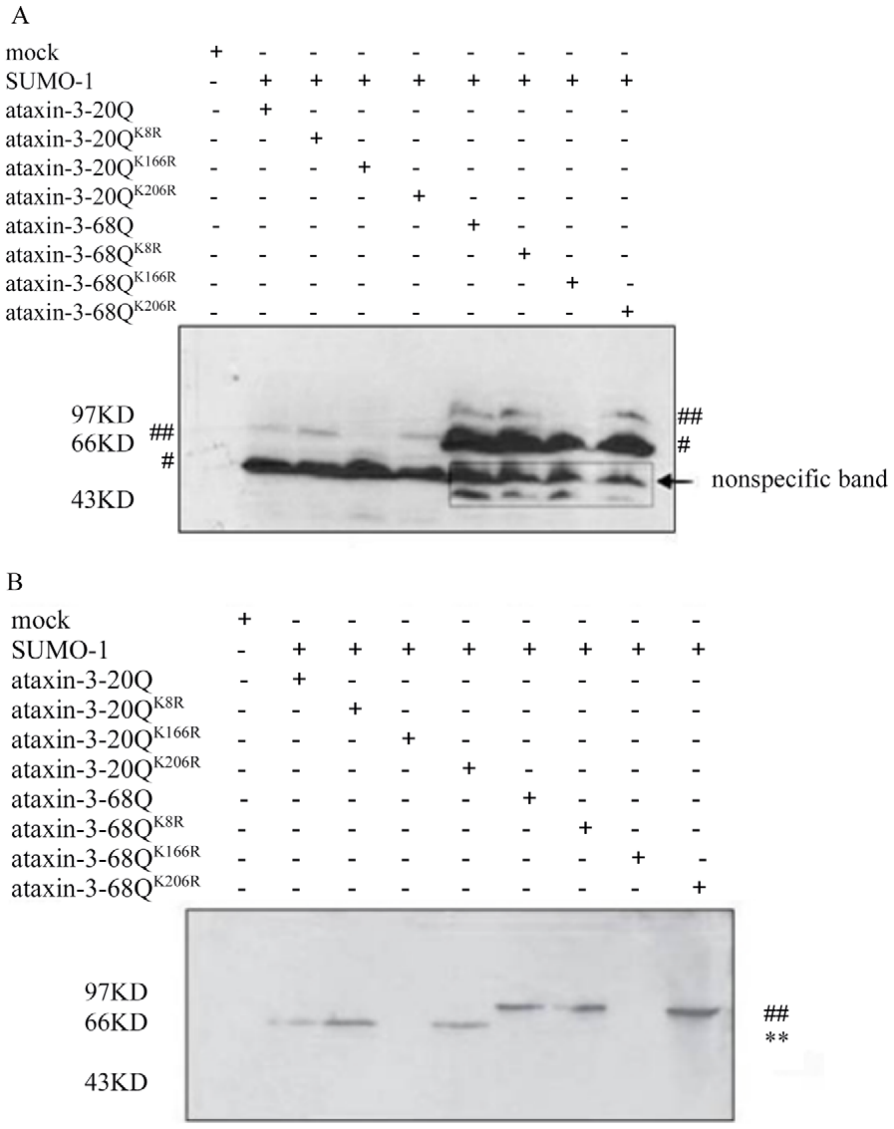
Identification of the SUMO-1 modification sites in ataxin-3. (A) HEK293 cells were used to co-express ataxin-3, ataxin-3^K8R^, ataxin-3^K166R^, or ataxin-3^K206R^ with SUMO-1. 10% lysates were precipitated by TCA and subjected to immunoblotting. #ataxin-3 main bands, ## ataxin-3 relative levels of modified and unmodified (whole-cell–TCA precipitates). (B) The tagged proteins were enriched with NTA magnetic nickel columns and detected by immunoblotting with SUMO-1 antibody. **, ataxin-3-20Q modified by SUMO-1, ##, ataxin-3-68Q modified by SUMO-1.

Firstly, the potential SUMOylation motifs on ataxin-3 were predicted by software, “SUMOplot™ prediction” (www.abgent.com/doc/sumoplot). The result suggested at least three consensus SUMOylation sequences in ataxin-3, which were K8 in EKQE, K166 in VKGD and K206 in HKTD. Based on these outputs, we constructed three mutants of ataxin-3, ataxin-3^K8R^, ataxin-3^K166R^, and ataxin-3^K206R^, in which the lysine 8, lysine 166 or lysine 206 were all converted to arginine (R). As shown in Figure 1, slow migrating bands were observed using both ataxin-3^K8R^ and ataxin-3^K206R^ as binding substrates of SUMO-1 while no migration was observed when ataxin-3^K166R^ was used. The results presented in Figure 1 clearly showed that only the conversion of lysine 166 to arginine abrogated the SUMOylation of ataxin-3, meaning lysine 166 was the SUMOylation site in ataxin-3.

### SUMO-1 modification of ataxin-3 did not affect its subcellular localization

**Figure 2:**
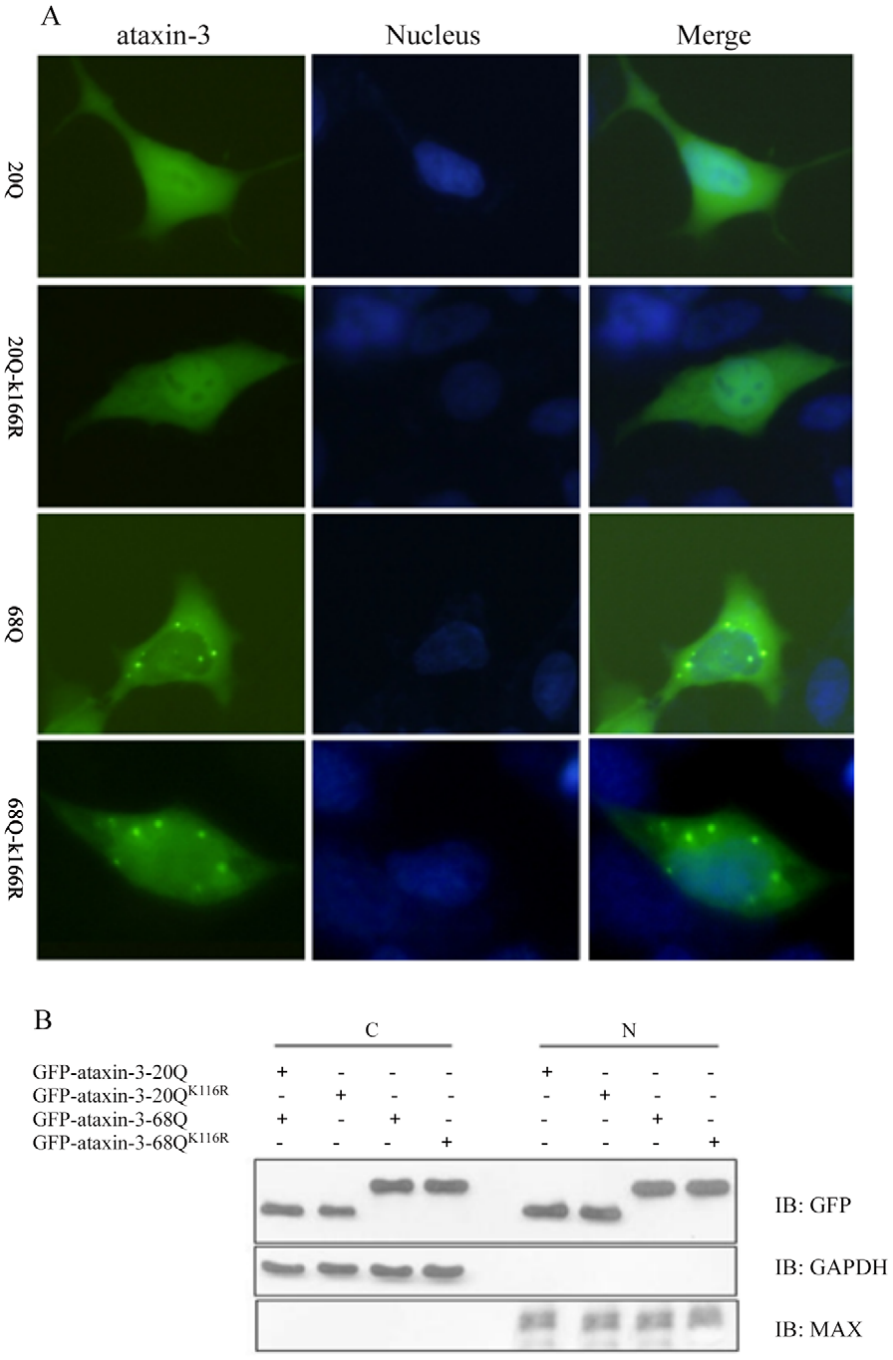
SUMO-1 modification did not affect the subcellular localization of ataxin-3. HEK293 cells were transfected with plasmids expressing GFP-tagged ataxin-3 or mutant ataxin-3^K166R^ in the presence of endogenous SUMO-1. Both ataxin-3-20Q and ataxin-3-20Q^K166R^ were localized in the nucleus and cytoplasm uniformly, and the aggregates that formed expressed ataxin-3-68Q and ataxin-3-68Q^K166R^ (A). Immunoblotting analysis of subcellular fractionation of ataxin-3 shows no differences between the various groups (B).

SUMOylation has been reported to be able to regulate the subcellular localization of several proteins. To examine whether SUMOylation of ataxin-3 affects its localization, we compared the localization of ataxin-3 and the SUMOylation deficient variant ataxin-3^K166R^ in transiently transfected HEK293 cells (Figure 2A). As previously described, overexpression of ataxin-3-20Q showed a diffusive distribution both in the nucleus and in the cytoplasm. The subcellular localization of ataxin-3-20Q^K166R^ was similar to that of ataxin-20Q. Interestingly, aggregates formation in overexpressed ataxin-3-68Q was also the same as that in ataxin-3-68Q^K166R^ transfected cells. There was significantly difference between wild-type and mutant type ataxin-3, the latter one formed aggregates but the former one did not (Figure 2A). The proteins levels in the fractions of cytoplasm and nucleus in the cells transfected with above plasmids suggested again that SUMO-1 modification did not affect subcellular localization of ataxin-3 (Figure 2B).

### SUMO-1 modification did not affect ataxin-3 ubiquitination or aggregate formation, but partially increased ataxin-3-68Q stability

**Figure 3:**
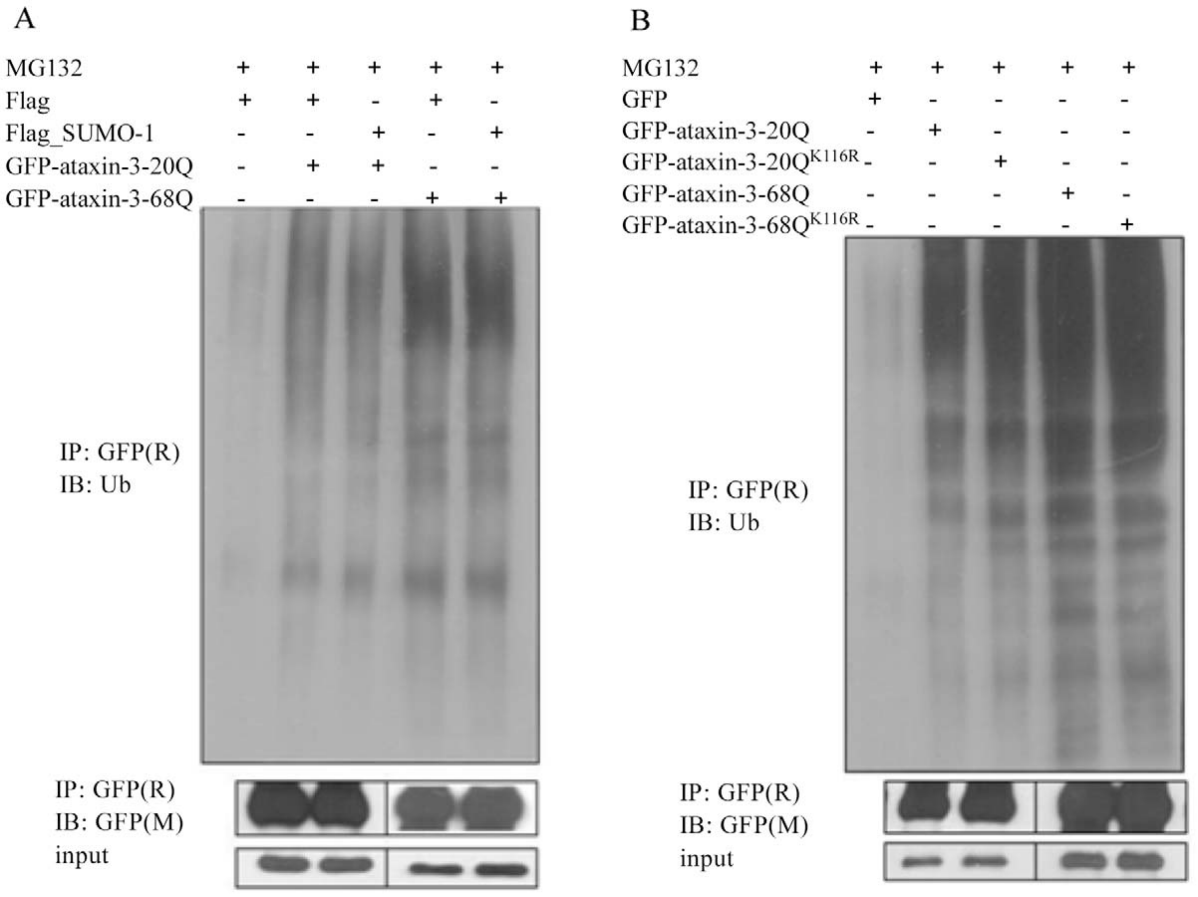
SUMO-1 modification did not affect ataxin-3 ubiquitination. (A) HEK293 cells were co-transfected with GFP-ataxin-3 and Flag-SUMO-1. The cells were treated with 10 µM MG132 for 12 h and subject to immunoprecipitation analysis using rabbit polyclonal antibodies against GFP. The immunoprecipitants were subject to immunoblotting analysis with the indicated antibodies. (B) HEK293 cells were transfected with GFP-ataxin-3 or GFP-ataxin-3^K166R^. The cells were treated with 10 µM MG132 for 12 h and subject to immunoprecipitation analysis using rabbit polyclonal antibodies against GFP. The immunoprecipitants were subject to immunoblotting analysis with the indicated antibodies.

It has been reported that SUMOylation alters the function or subcellular localization of some proteins, and the competition between SUMO-1 and ubiquitin for identical binding sites protects some proteins from degradation [33]. To determine whether SUMO-1 modification would affect the ubiquitination of ataxin-3, we transiently expressed GFP-ataxin-3 or GFP-ataxin-3^K166R^ in HEK293 cells and performed immunoprecipitation assays using anti-GFP antibodies. The ubiquitination of ataxin-3 and ataxin-3^K166R^ was not significantly different, which suggested that SUMO-1 modification did not affect the ubiquitination of ataxin-3, and lysine 166 might not be the ubiquitination site (Figure 3A, 3B).

**Figure 4:**
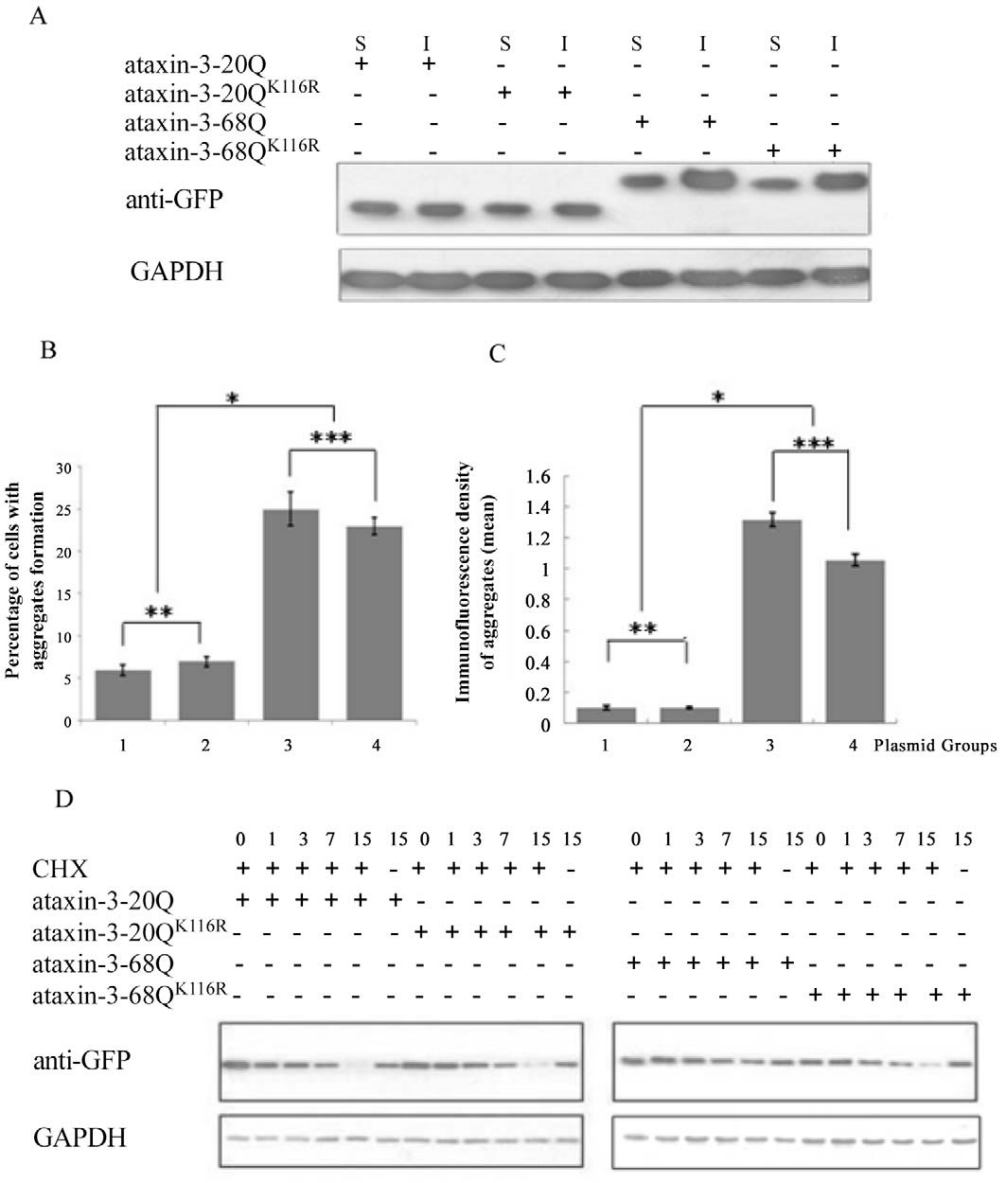
SUMO-1 modification partially increased ataxin-3-68Q stability. HEK293 cells were transfected with GFP-ataxin-3 or GFP-ataxin-3^K166R^. Immunoblotting analysis showed difference between the soluble (S) and insoluble (I) ataxin-3 in 20Q and 68Q with or without K166 (A). At 48 h after transfection, both aggregates formation cells and its immunoflurescence density were quantified. Plasmid groups: 1. GFP-ataxin-3-20Q; 2. GFP-ataxin-3-20Q^K166R^; 3. GFP-ataxin-3-68Q; 4. GFP-ataxin-3-68Q^K166R^. Statistical significance was assessed with a one-way ANOVA. The amount of aggregates formation cells: 1 and 3: P<0.05 (*); 1 and 2: P>0.05 (**); 3 and 4: P>0.05 (***) (B). Immunoflurescence density of aggregates: 1 and 3: P<0.05 (*); 1 and 2: P>0.05 (**); 3 and 4: P>0.05 (***) (C). At 24 h after transfection, cells were treated with CHX (100 µg/ml) to prevent protein synthesis. Cells were harvested at 0, 1, 3, 7, 15 h after CHX treatment, subject to 12% SDS-PAGE, and analyzed by immunoblotting with anti-GFP antibody (D).

Since SUMO modification may regulate the stability of proteins [33]–[34], we speculated that SUMO-1 modification might alter the stability of ataxin-3. The levels of sumoylated and un-sumoylated proteins were examined in cells transfected with ataxin-3 or ataxin-3^K166R^. Firstly, we detected the soluble and insoluble fractions of cell lysate by western blot separately. The results showed that the bands of insoluble fraction of mutant-type ataxin-3 were stronger than that of the wild-type, which suggested that stabilized mutant ataxin-3 led to aggregate formation and induced the disease of SCA3/MJD. In addition, both bands of soluble and insoluble fraction of ataxin-3-68Q were denser than those of ataxin-3-68Q^K166R^, indicating SUMOylation might increase the stability of ataxin-3-68Q (Figure 4A). Subsequently, we investigated whether the enhanced protein fraction of sumoylated ataxin-3-68Q was related with the increased aggregate formation. To address this possibility, we quantified aggregate formation cells and immunoflurescence density of aggregates by fluorescence imaging and imageJ computational analysis. Unfortunately, there was no significant difference existed between either ataxin-3-20Q and ataxin-3-20Q^K166R^ or ataxin-3-68Q and ataxin-3-68Q^K166R^ (P>0.05) (Figure 4B, 4C). Finally, the chase experiment was used to understand the effect of SUMOylation on degradation of ataxin-3. As shown in Figure 4D and Figure S1, the level of ataxin-3-68Q was significantly higher than that of ataxin-3-68Q^K166R^ especially at 15 h after CHX treatment, while the level of ataxin-3-20Q was similar to that of ataxin-3-20Q^K166R^. These data suggested that SUMOylation of ataxin-3-68Q might partially enhance the stability of ataxin-3-68Q.

### SUMO-1 modification increased cytotoxicity of ataxin-3-68Q

**Figure 5:**
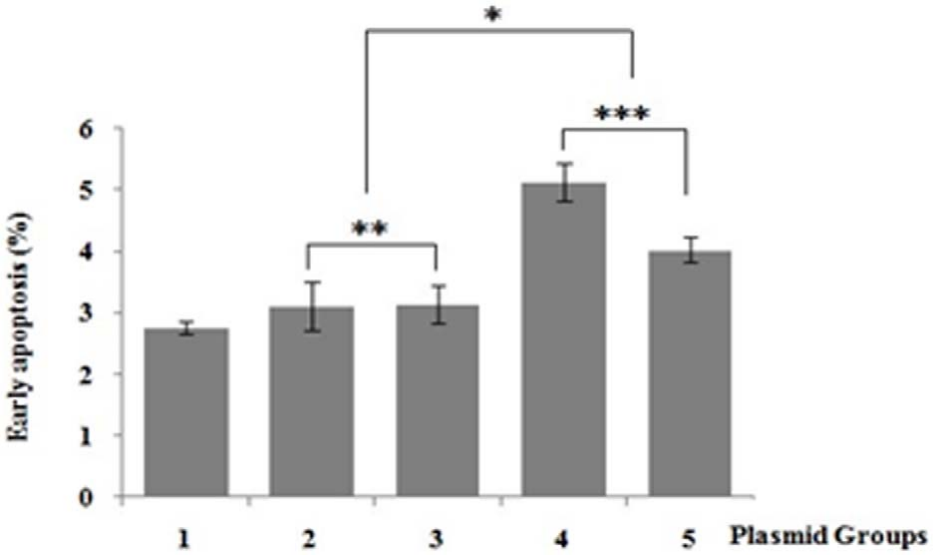
Early apoptosis rate in HEK293 cells. Plasmid Groups: 1. pcDNA3.1-myc-His(-)B; 2. pcDNA3.1-myc-His(-)B-ataxin-3-20Q; 3. pcDNA3.1-myc-His(-)B-ataxin-3-20Q^K166R^; 4. pcDNA3.1-myc-His(-)B-ataxin-3-68Q; 5. pcDNA3.1-myc-His(-)B-ataxin-3-68Q^K166R^. Statistical significance was assessed with a one-way ANOVA: 2 and 4: P<0.05 (*); 2 and 3 P>0.05 (**); 4 and 5: P<0.05 (***).

We examined the cytotoxicity effects by flow cytometry analysis using PI/Annexin V-FITC staining in HEK293 cells transfected with myc-ataxin-3 or myc-ataxin-3^K166R^. Relatively high percentages of early apoptosis rate were found in ataxin-3-68Q transfected cells compared to that of ataxin-3-68Q^K166R^ transfected ones (P<0.05), suggesting SUMO-1 modification might had a cytotoxic effect. However, there was no significant difference between the early apoptosis rates of ataxin-3-20Q and that of ataxin-3-20Q^K166R^ group (P>0.05) (Figure 5).

## Discussion

Recent studies have revealed that some neurodegenerative disease proteins, such as androgen receptor (AR) [25], huntingtin [26], ataxin-1 [27], ataxin-7 [28], DJ-1 [35], tau [36], and a-synuclein [36], are also modified by SUMOs, implying that SUMOylation of these disease-related proteins may participate in the regulation of their functions and thereby be associated with their pathogenic role. Tau, an Alzheimer's disease associated protein, has been reported to be SUMOylated by SUMO-1, and to a much lesser extent by SUMO-2 or SUMO-3 simultaneously [36]. Many substrates were reported to be multi-SUMOylated by SUMO-1 on several residues, for example, ataxin-1 modified at least on five and huntingtin on three lysine residues [26]–[27]. SCA3/MJD is the most common spinocerebellar ataxia diseases. In our previous research, we found that ataxin-3 was also a substrate of SUMO-1 [32]. In order to identify the motif residue, mutagenesis analyses were carried out to converse lysine 166 residues to arginine, which lies within a SUMO consensus sequence, VKGD, in ataxin-3. This conversion completely blocked the SUMOylation of ataxin-3. However, the conversion of other lysines, K8 and K206, which also lie within the SUMO consensus sequence in ataxin-3, did not affect SUMOylation of ataxin-3. These data suggest that K166 in ataxin-3 is the major SUMOylation binding site.

Modification by SUMO has been shown to play critical roles in subcellular localization, and protein degradation, which ultimately contribute to regulation of the cell cycle, cell growth, and apoptosis [37]. In order to examine whether SUMOylation of ataxin-3 affects its subcellular localization, we compared the localization of ataxin-3 in transiently transfected HEK293 cells. In agreement with previous studies, we found that the wild-type ataxin-3 protein was diffusively distributed in both nucleus and cytoplasm, while mutant-type ataxin-3 protein formed aggregates in nucleus. However, when we compared ataxin-3 and its SUMOylation deficient variant, we could not detect any difference in the subcellular localization of ataxin-3 in both immunofluorescent staining and immunoblot analysis, which indicates SUMOylation of ataxin-3 does not change its subcellular distribution. The similar result was also observed in SCA7, that SUMOylation on K257 of ataxin-7 does not influence its subcellular localization [28].

As we know, abnormal accumulation of mutant ataxin-3 in affected neurons reflexes that mutant protein may not be properly degraded. We found the insoluble fraction of ataxin-3-68Q was more than that of ataxin-3-20Q, which supported that mutant-type ataxin-3 protein was stable and easy to form aggregates. As SUMO modification of proteins is involved in protein degradation, it is possible that sumoylation of ataxin-3 may regulate its degradation process. Since SUMO-1 modifications target the same lysine residue as ubiquitin, many researches have revealed a dynamic interplay between the related ubiquitination and SUMOylation pathways [38]. We first performed immunoprecipitation assays to detect the ubiquitination differences between ataxin-3 and ataxin-3^K166R^. However, we didn't find any evidence that SUMOylation of ataxin-3 affect ataxin-3 ubiquitination, which also indicate there is no competition between SUMO-1 and ubiquitin for binding site K166.

Subsequently, the soluble/insoluble and total protein level of sumoylated and un-sumoylated proteins were also examined, both bands of soluble and insoluble fraction of ataxin-3-68Q were denser than those of ataxin-3-68Q^K166R^ indicating the SUMOylation modification of mutant-type ataxin-3 might enhance the stability of the protein and participate in the pathogenesis process of SCA3/MJD to a certain degree. In addition, we further confirmed SUMO-1 modification decreased the degradation and enhanced the stability of mutant-type ataxin-3 by chase assay. Therefore, we have no reason to doubt that although SUMO-1 modification on K166 does not influence the UPS pathway but probably affect other processes such as autophagy for mutant-type ataxin-3 degradation. Increased polyQ-expanded ataxin-3 stability might leads to multiple consequences. On the one hand, polyQ-expanded ataxin-3 is more easily gathered to form aggregates. On the other hand, the concentration of the monomer or oligomer of polyQ-expanded ataxin-3 might increases as huntingtin (26), leading to increased cytotoxicity, promotion of apoptosis, and acceleration of the pathological process in SCA3/MJD pathogenicity.

PolyQ disorders are characterized pathologically by the accumulation of protein aggregates within neurons. Whether the microscopically visible inclusions play a causal role in disease pathogenesis or protect neurons from the affects of toxic proteins remains unclear [26], [39]. Therefore, as a central pathological event in polyQ disorders, aggregation needs to be better understood, particularly from a therapeutic point of view. In agreement with previous studies [40], we found the amount of aggregate formation cells in mutant-type ataxin-3 as much higher than that in normal control; demonstrating polyQ expansion could induce the formation of aggregates. Although there was no significantly difference in both aggregate cell counting and density quantification between ataxin-3-68Q and ataxin-3-68Q^K166R^, we could found the tendency that aggregate density of ataxin-3-68Q was slightly higher than that of ataxin-3-68Q^K166R^, which support the results of insoluble fraction detection and indicate that SUMOylation of mutant-type ataxin-3 might partially increase its stability and probably promote aggregate formation.

It has been reported that protein aggregates could sequester polyQ proteins which affects their normal biological function [39] and finally result in polyQ diseases. SUMOylation of the polyQ proteins might influences their aggregation and toxicity. For example, SUMOylation of the polyQ-expanded AR decreases the amount of the SDS-insoluble aggregates [41], and study on huntingtin proposed that SUMOylation may explain the intriguing cell death observed in polyQ disorders [42]. As what we show in Figure 5, SUMO-1 modification of mutant-type ataxin-3 increased the early apoptosis rate of the neurons, indicating that SUMOylation might enhance the stability of mutant-type ataxin-3, thus increase its cytotoxicity, however the concrete mechanism still needs intensive study in future.

In conclusion, our study demonstrated that SUMOylation on K166, the first described residue of SUMO-1 modification of ataxin-3, partially increased the stability of mutant-type ataxin-3, and the rate of apoptosis arisen from the cytotoxicity of the modified protein. Those support the hypothesis that SUMO-1 modification has a toxic effect on mutant-type ataxin-3 and participates in the pathogenesis of SCA3/MJD. Further studies in *Drosophila* models should be done to confirm these findings.

## Materials and Methods

### Plasmid construction

Plasmids for myc-ataxin-3 and SUMO-1 in pcDNA3.1-myc-His(-)B (Invitrogen) have been described previously [32]. Ataxin-3^K8R^, ataxin-3^K166R^, and ataxin-3^K206R^ were all generated by site-directed mutagenesis using long primers and overlap methods with primers M1/M2, M3/M4, M5/M6, respectively. GFP-ataxin-3 and GFP-ataxin-3^K166R^ were constructed by subcloning the PCR product amplified using primers M1/M2 with pcDNA3.1-myc-His(-) B-ataxin-3 into pEGFP-N1 (Invitrogen) at *Sal*I/*Bam*HI sites respectively. The p3×FLAG-myc-CMV-24-SUMO-1 plasmid was kindly provided by Professor Wang Guanghui. All constructs were confirmed by sequencing. Primers used in this study are shown in Table 1.

**Table 1:** Primers for amplification.

Primers*	Sequence
W1	5′-ACGGGATCCGCCACCATGGAGTCCATCTTCCACG-3′
W2	5′-CCCAAGCTTGGGCATGTCAGATAAAGTGTGAAGG-3′
M1	5′-ACGGGATCCGCCACCATGGAGTCCA-3′
M2	5′- ATCTTCCACGAGAGACAAGGTACG-3′
M3	5′-TTTGTTGTTAGAGGTGATCTGCCAG-3′
M4	5′-CAGATCACCTCTAACAACAAATATAG-3′
M5	5′-AGAGTCCATAGAACAGACCTGGAACG-3′
M6	5′-AGGTCTGTTCTATGGACTCTTTGCTC-3′

* Primers used are described in Experimental Procedures.

### Cell culture and transfection

HEK293 cells were cultured overnight in Dulbecco's modified Eagle's medium (DMEM) (Gibco) supplemented with 10% fetal bovine serum (FBS) (Gibco) and antibiotics penicillin/streptomycin at 37°C under 5% CO_2_, and then transfected with expressing plasmids using Lipofectamine™ 2000 reagent (Invitrogen) according to the manufacturer's protocol in DMEM without FBS. The same volume of DMEM containing 10% FBS was added to the culture medium 6 h after transfection. Forty eight hours after transfection, the transfected cells were observed using an inverted system microscope IX71 (Olympus) or used for immunofluorescent staining, immunoblot analysis, or co-immunoprecipitation.

### Preparation of cell extracts and NTA precipitation

Thirty hours after transfection, cells were lysed in 1 ml of lysis buffer (6M guanidine hydrochloride, 100 mM NaH_2_PO_4_, and 10 mM Tris [pH 7.8]). After sonication, 90% lysate was incubated with 25 µl of Ni–nitrilotriacetic acid (NTA) magnetic agarose beads (Qiagen). The beads were washed twice with washing buffer (pH 7.8) containing 8 M urea, followed by washing with a buffer (pH 6.3) containing 8 M urea. After a final wash with phosphate-buffered saline (PBS), the beads were eluted with 2×SDS sample buffer for immunoblot analysis. Then 10% lysate was subjected to trichloroacetic acid (TCA) precipitation and used as a whole cell extract (WCE). The proteins were analyzed by Western blotting using the appropriate antibodies as described recently [33].

### Fluorescence

HEK293 cells were plated onto cover slips in a 12-well plate. The following day they were transfected using Lipofect2000™ (Invitrogen). Forty-eight hours after transfection, they were incubated 10 µg/ml Hoechst 33258 (Sigma) to visualize the nucleus for 5 min at 37°C. Analysis was performed using an inverted system microscope IX71 (Olympus).

### Subcellular fractionation

HEK293 cells transfected with expression plasmids were fractionated into cytoplasmic and nuclear fractions 24 h after transfection. After being washed twice with pre-cold PBS, cells were lysed in fractionation buffer containing 10 mM Tris-HCl (pH 7.5), 1 mM EDTA, 0.5% NP-40 and complete mini protease inhibitor cocktail, for 30 min at 4°C. Following centrifugation at 600×g for 10 min at 4°C, the supernatant was collected as the cytoplasmic fraction. The pellets, resuspended with pellet buffer containing 2% SDS, as the nuclear fraction.

### Immunoprecipitation

HEK293 cells were collected 48 h after transfection. The cells were sonicated in TSPI buffer (50 mM Tris-HCl [pH 7.5], 150 mM sodium chloride, 1 mM EDTA, 1 µg/ml of aprotinin, 10 µg/ml of leupeptin, 0.5 µM Pefabloc SC, and 10 µg/ml of pepstain) containing 1% NP-40. Cellular debris was removed by centrifugation at 12,000×g for 15 min at 4°C. The supernatants were incubated with the antibodies in 0.01% BSA for 4 h at 4°C. After incubation, protein G Sepharose (Roche) was used for precipitation. The beads were washed with TSPI buffer four times, and then bound immunoprecipitants were eluted with 2×SDS sample buffer for immunoblot analysis.

### RIPA-soluble and RIPA-insoluble fraction

For serial extraction in RIPA and formic acid, cells were washed twice in PBS and then lysed in 600 µl RIPA buffer and centrifuged for 20 min at 40,000 g at 4°C. Supernatant was collected as the soluble protein for Western blot, and the pellet was resuspended in 100 µl 70% formic acid with sonication until clear. Formic acid samples were then neutralized by adding 1.9 ml 1 M Tris base and diluted 1∶3 in H_2_O as the insoluble protein for Western blot.

### Immunoblot analysis

Proteins were separated by 10%/12% SDS–PAGE and then transferred onto polyvinylidene difluoride membrane (Millipore). The following primary antibodies were used: monoclonal anti-c-myc antibody (Sigma), monoclonal anti-SUMO-1 antibody (Zymed), monoclonal anti-GFP antibody (Santa Cruz), monoclonal anti-ubiquitin antibody (Santa Cruz), monoclonal anti-GAPDH (Chemicon). Sheep anti-mouse IgG-HRP antibody was used as the secondary antibody. The proteins were visualized using an ECL detection kit (Amersham Pharmacia Biotech).

### Quantitation of aggregates and immunoflurescence density of aggregates

HEK293 cells were transfected with plasmids expressing GFP-tagged ataxin-3 or mutant ataxin-3^K166R^. Forty-eight hours after transfection, the transfected cells were observed using an inverted system microscope IX71 (Olympus). All cells were counted in fields selected at random from five different quadrants of the culture well. Counting was performed by an investigator blind to the experimental conditions. By ImageJ software, the immunoflurescence density of aggregates (mean) was measured. The assay was carried out in triplicate.

### Degradation assay

HEK293 cells were transiently transfected with GFP-ataxin-3 and GFP-ataxin-3^K166R^. Twenty four hours after transfection, cells were treated with 100 µg/ml cycloheximide (CHX) (Sigma) to inhibit protein synthesis and were harvested at 0, 1, 3, 7, 15 h or 0, 15, 15 h after CHX treatment. The same volume of lysates was analyzed by immunoblotting using anti-GFP antibody (Santa Cruz).

### PI/Annexin V-FITC double-stained flow cytometry

HEK293 cells were transfected with expressing vectors. Forty eight hours after transfection, apoptosis were assessed by using FACSCalibur flow cytometer (Becton Dickinson) according to the manufacturer's instructions. Briefly, cells were collected by centrifugation in a refrigerated microcentrifuge and resuspended with 200 µl binding buffer. After the addition of 10 µl annexin V-fluorescein isothiocyanate (FITC) and 5 µl propidium iodide (PI), cells were kept in dark at room temperature for 15 min. Then the cells were added to another 300 µl binding buffer and assessed within 1 h. Data analysis was performed with CellQuest software.

## Supporting Information

Figure S1 **SUMO-1 modification increases ataxin-3-68Q stability. HEK293 cells were transfected with GFP-ataxin-3 or GFP-ataxin-3^K166R^.** At 24 h after transfection, cells were treated with CHX (100 µg/ml) to prevent protein synthesis. Cells were harvested at 0, 15 h after CHX treatment, subject to 12% SDS-PAGE, and analyzed by immunoblotting with anti-GFP antibody.
(TIF)
