# Supplementary figures and images for: SUMO-1 Modification on K166 of PolyQ-Expanded aTaxin-3 Strengthens Its Stability and Increases Its Cytotoxicity
Source: PLoS One. 2013 Jan 31;8(1):e54214. doi: 10.1371/journal.pone.0054214 (PMC3561348; doi:10.1371/journal.pone.0054214)

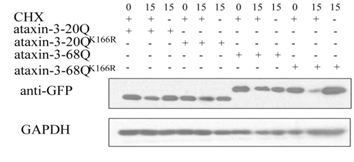

Supplement: Figure S1 — SUMO-1 modification increases ataxin-3-68Q stability. HEK293 cells were transfected with GFP-ataxin-3 or GFP-ataxin-3K166R. At 24 h after transfection, cells were treated with CHX (100 µg/ml) to prevent protein synthesis. Cells were harvested at 0, 15 h after CHX treatment, subject to 12% SDS-PAGE, and analyzed by immunoblotting with anti-GFP antibody. (TIF) [file pone.0054214.s001.tif]
